# Supplementary material for: Ferroptotic cell death triggered by conjugated linolenic acids is mediated by ACSL1
Source: Nat Commun. 2021 Apr 14;12:2244. doi: 10.1038/s41467-021-22471-y (PMC8046803; doi:10.1038/s41467-021-22471-y)
Supplement: Supplementary file 1 — Supplementary Information [file 41467_2021_22471_MOESM1_ESM.pdf]

Supplementary Figure 1

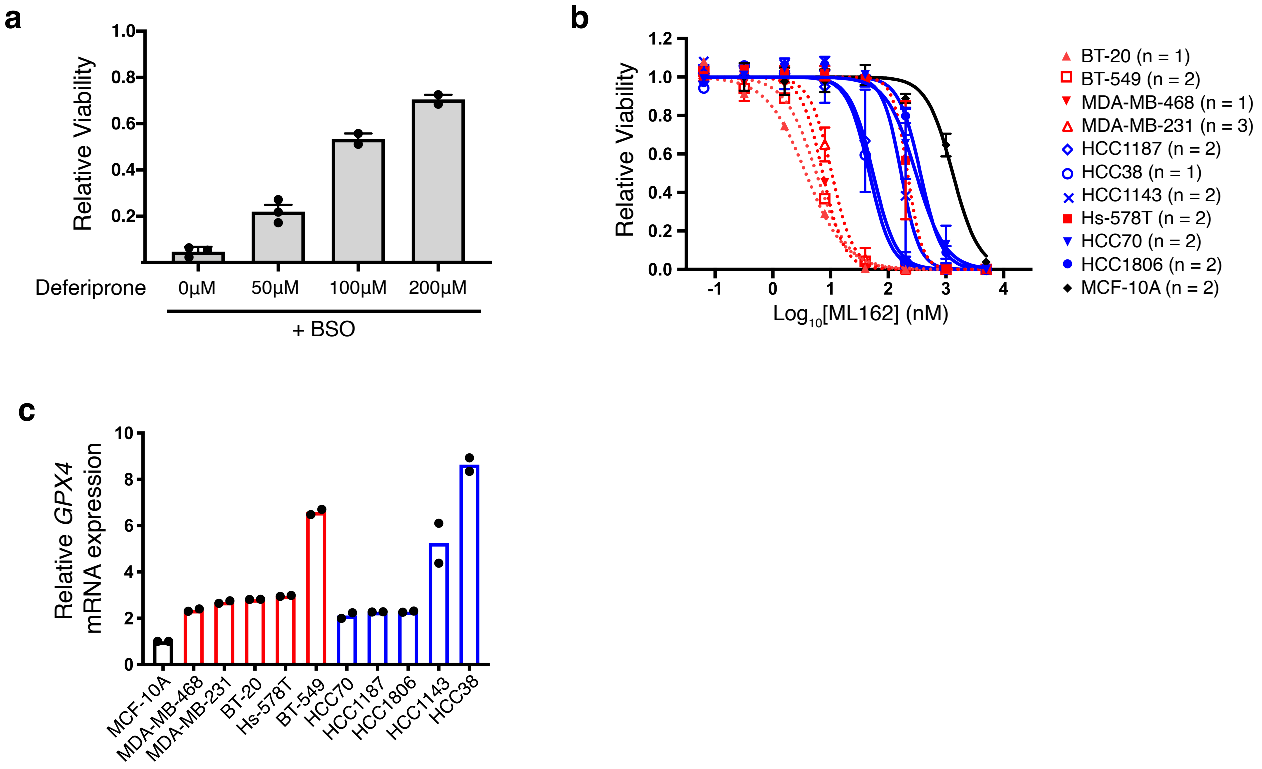

Supplementary Figure 1. **A subset of TNBC cells undergo ferroptosis following glutathione depletion and are sensitive to GPX inhibition.** **a**, Relative viability of BT-549 cells incubated with 10 μM BSO and the specified dose of deferiprone for 72 hours (n = 3 independent experiments for the 0 and 50 μM deferiprone doses and n = 2 independent experiments for the 100 and 200 μM doses). Values were normalized to account for loss of viability associated with deferiprone. Error bars in this and the subsequent panels show standard deviation centered on the mean. **b**, Cell viability dose response curves for ML162 in 10 TNBC cells lines and the non-transformed mammary epithelial cell line MCF-10A after 72 hours of treatment. **c**, Relative mRNA expression of GPX4 measured by qPCR in the indicated cell lines (n = 2 technical replicates). In **b** and **c**, non-fer-1 suppressible and fer-1 suppressible TNBC cell lines (as defined in **Fig 1f**) are colored blue and red, respectively.

Supplementary Figure 2

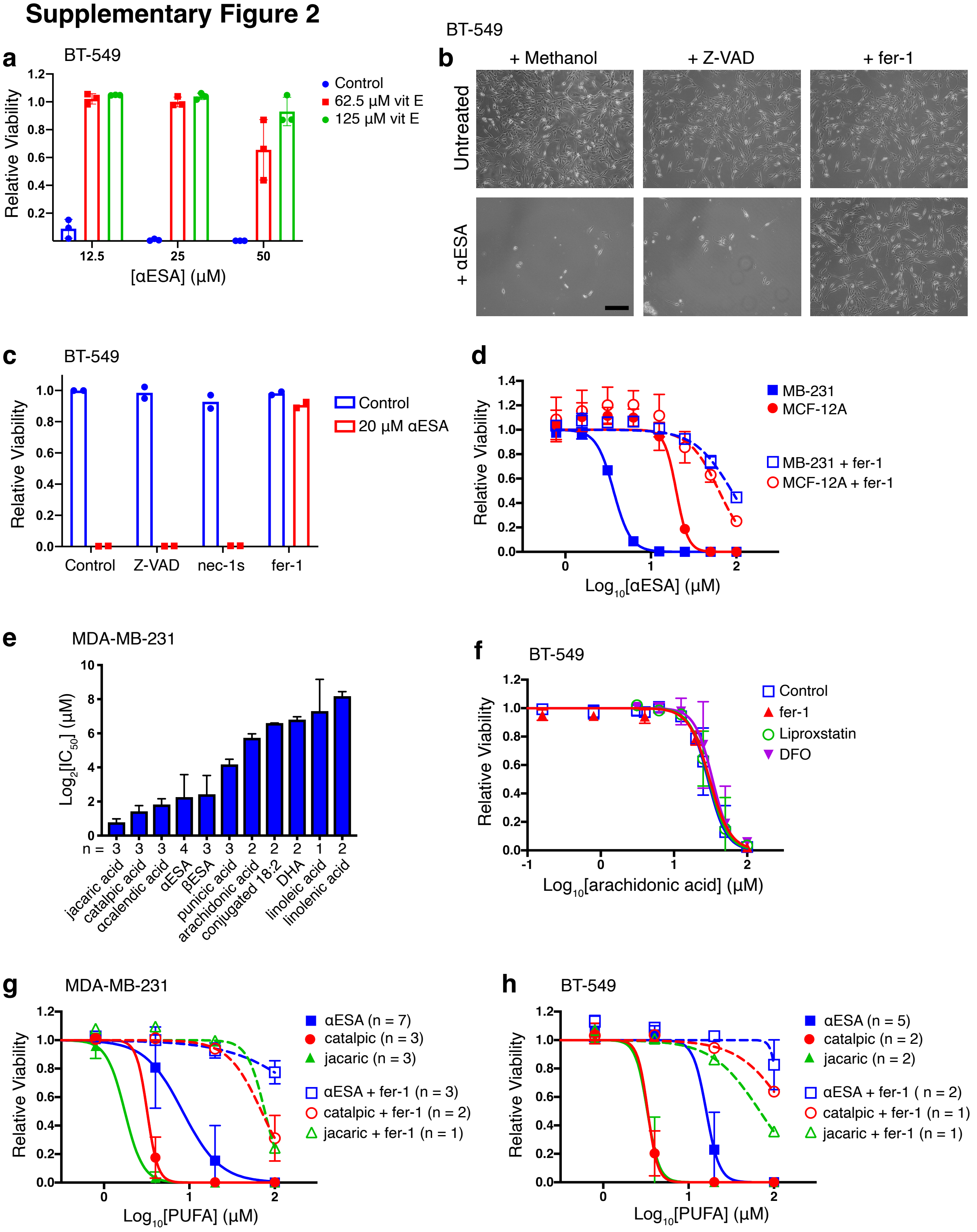

Supplementary Figure 2. **Conjugated linolenic acids trigger ferroptosis in TNBC cells. a**, Relative viability of BT-549 cells co-treated with the specified dose of *α*ESA and vitamin E (*α*-tocopherol) for 48 hours (n = 3 independent experiments). Error bars denote standard deviation centered on the mean unless otherwise noted. **b**, Light micrographs of BT-549 cells treated with 25 μM *α*ESA for 48 hours in the presence or absence of 20 μM Z-VAD or 2 μM fer-1. Scale bar represents 200 μm. This experiment was performed twice. **c**, Relative viability of BT-549 cells incubated for 72 hours with vehicle or 20 μM *α*ESA and either 20 μM Z-VAD, 50 μM nec-1s, or 2 μM fer-1 (n = 2 independent experiments). **d**, Relative cell viability dose response curves for *α*ESA in MDA-MB- 231 and MCF-12A with and without 2 μM fer-1 (n = 3 independent experiments). **e**, $Log_2(IC_{50})$ values for the indicated polyunsaturated fatty acid in MDA-MB-231 after 72 hours of incubation. Error bars show standard error of the mean for IC_{50} based on the fit of the dose-response curve. DHA = docosahexaenoic acid. **f**, Cell viability dose response curves for arachidonic acid in BT- 549 after 72 hours of treatment with or without 2 μM fer-1, 2 μM liproxstatin, or 50 μM DFO (n = 3 independent experiments for each condition). Cell viability dose response curves for the noted conjugated linolenic acid in the presence or absence of 2 μM fer-1 in **g**, MDA-MB-231 or **h**, BT- 549 cells. Treatment time was 48 hours for BT-549 and 72 hours for MDA-MB-231.

Supplementary Figure 3

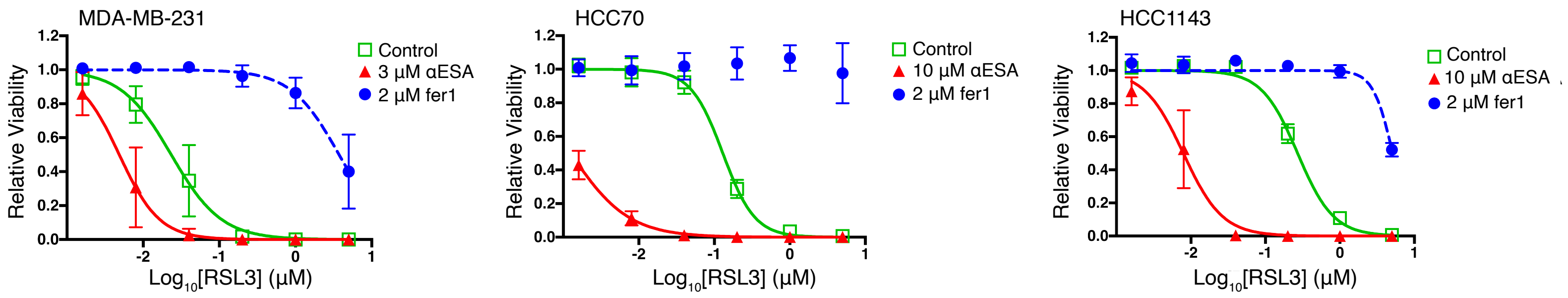

Supplementary Figure 3. **RSL3 toxicity is enhanced by αESA in TNBC cells.** Relative cell viability dose response curves for RSL3 in MDA-MB-231 (n = 4 independent experiments), HCC70 (n = 3), HCC1143 (n = 3) cells co-treated with either vehicle, the specified dose of αESA, or 2 μM fer-1. Cells were treated for 72 hours. Error bars show standard deviation centered on the mean.

Supplementary Figure 4

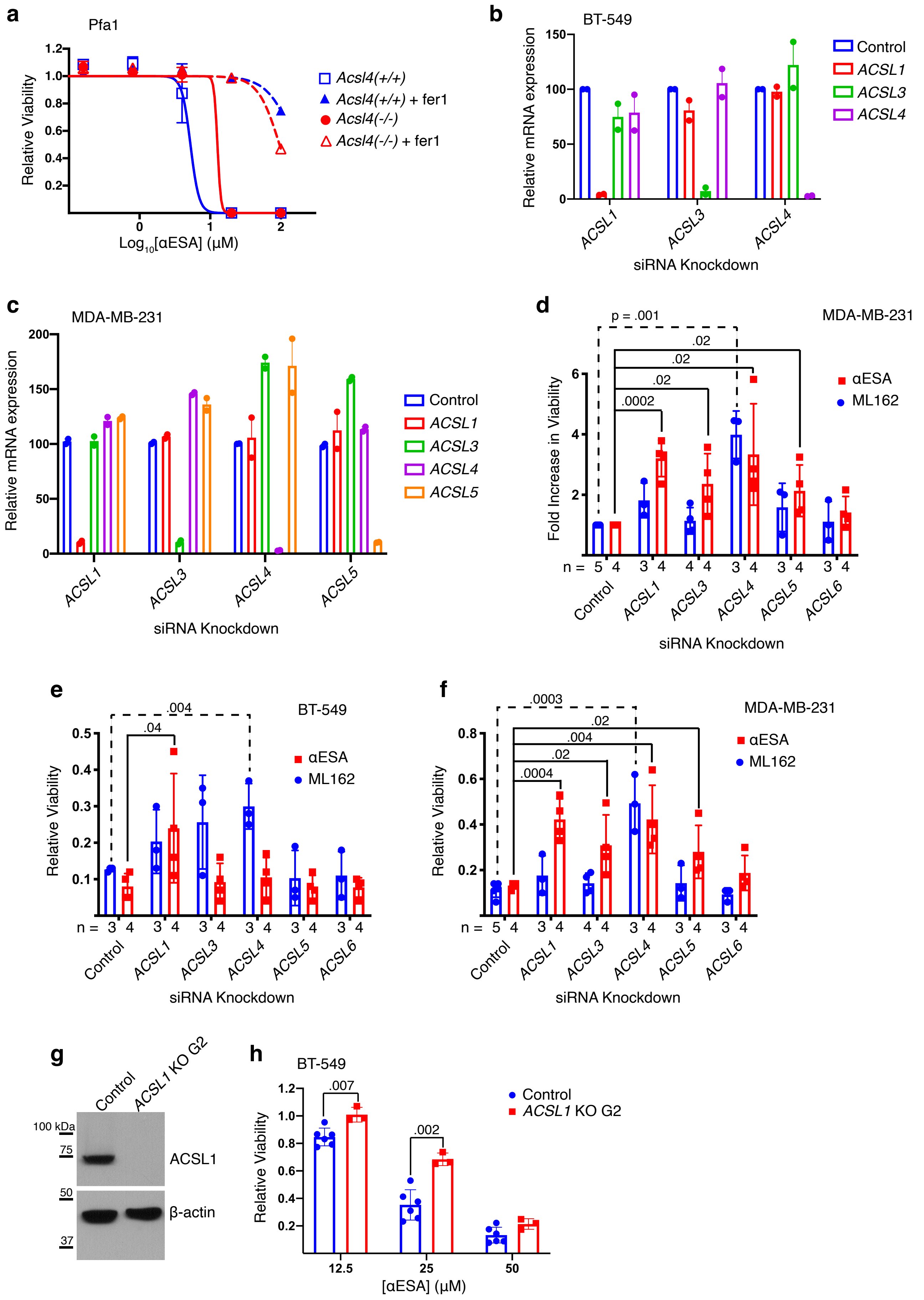

Supplementary Figure 4. **Mechanism of ferroptosis by *α*ESA.** **a**, Cell viability dose response curves for *α*ESA in control and *Acsl4*-deficient mouse embryonic fibroblast (Pfa1) cells with or without 2 μM fer- 1 (n = 2 independent experiments). Error bars in this and subsequent panels show standard deviation centered on the mean. Relative mRNA expression of ACSL family members by qPCR in **b**, BT-549 or **c**, MDA-MB-231 cells 72 hours after transfection with the indicated *ACSL* siRNA (n = 2 biological replicates). BT-549 do not express *ACSL5* and neither BT-549 nor MDA-MB- 231 express *ACSL6* at a level that could be quantified. **d**, Fold change in the fraction of viable MDA-MB-231 cells 72 hours after transfection with the noted *ACSL* siRNA followed by 24 hours of treatment with either ML162 (blue) or *α*ESA (red). Viability fold change is relative to cells transfected with a non-targeting siRNA. p values (Student's t-test, one-sided) below the significance threshold are shown. **e**, Relative viability values corresponding to the data presented in Figure 5c and **f**, relative viability values corresponding to the data presented in Figure S4d. p values (Student's t-test, one-sided) below the significance threshold are shown. **g**, Western blot showing ACSL1 protein levels in control BT-549 cells expressing a non-targeting guide RNA and a clone of BT-549 derived from a single cell in which *ACSL1* was mutated using CRISPR/Cas9 (*ACSL1* KO G2 L1). The guide used to generate this line is distinct from the guide used to make the lines described in Figure 5d, e. *β*-actin is the loading control. **h**, Relative cell viability of control (n = 6) and *ACSL1* KO cells (n = 3) after 48 hours of treatment with the specified dose of *α*ESA. The p-values above the comparator bars are from a two-sided Student's t-tests.

Supplementary Figure 5

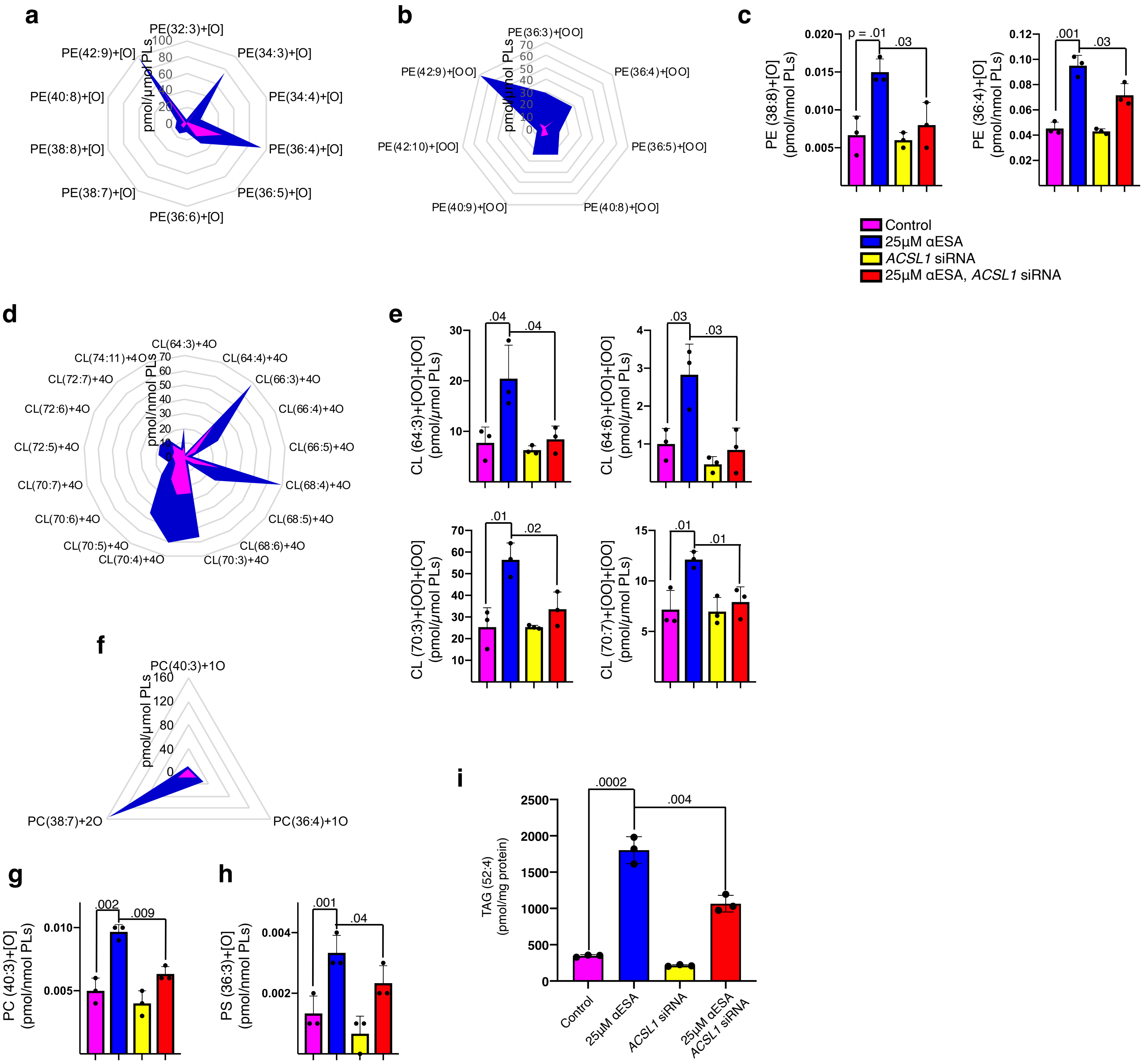

Supplementary Figure 5. **Quantitative LC/MS assessment of oxidized phospholipids altered by αESA treatment in BT-549 cells.** Radar plot showing significant increases in the amount of **a**, mono- and **b**, di-oxygenated PE species after treatment with 25 μM αESA for 8 hours. Amount of phospholipid is shown in magenta for controls and blue for αESA-treated cells for all radar plots. **c**, Bar charts showing the amounts of PE species that were significantly increased by αESA treatment compared to vehicle-treated controls and decreased in αESA-treated cells in which ACSL1 had been depleted compared to αESA-treated cells. Data in this and subsequent bar charts are based on three biological replicates and error bars show standard deviation centered on the mean. The color scheme shown in this panel applies to subsequent bar charts. p-values are shown above the comparator bars (Student's t-test, two-sided) **d**, Radar plot showing significant increases in oxidized CL species after treatment with αESA. **e**, Bar charts showing the amountsof oxidized CL species that were significantly increased by αESA treatment and responsive to ACSL1 knockdown. **f**, Radar plot showing significant increases in oxidized PC species after treatment with αESA. Amounts of **g**, a PC species and **h**, a PS species that were significantly increased by αESA treatment and decreased by ACSL1 depletion. **i**, Bar chart showing the amount of a TAG (52:4) species that was significantly increased by αESA treatment in an ACSL1-dependent manner.

Supplementary Figure 6

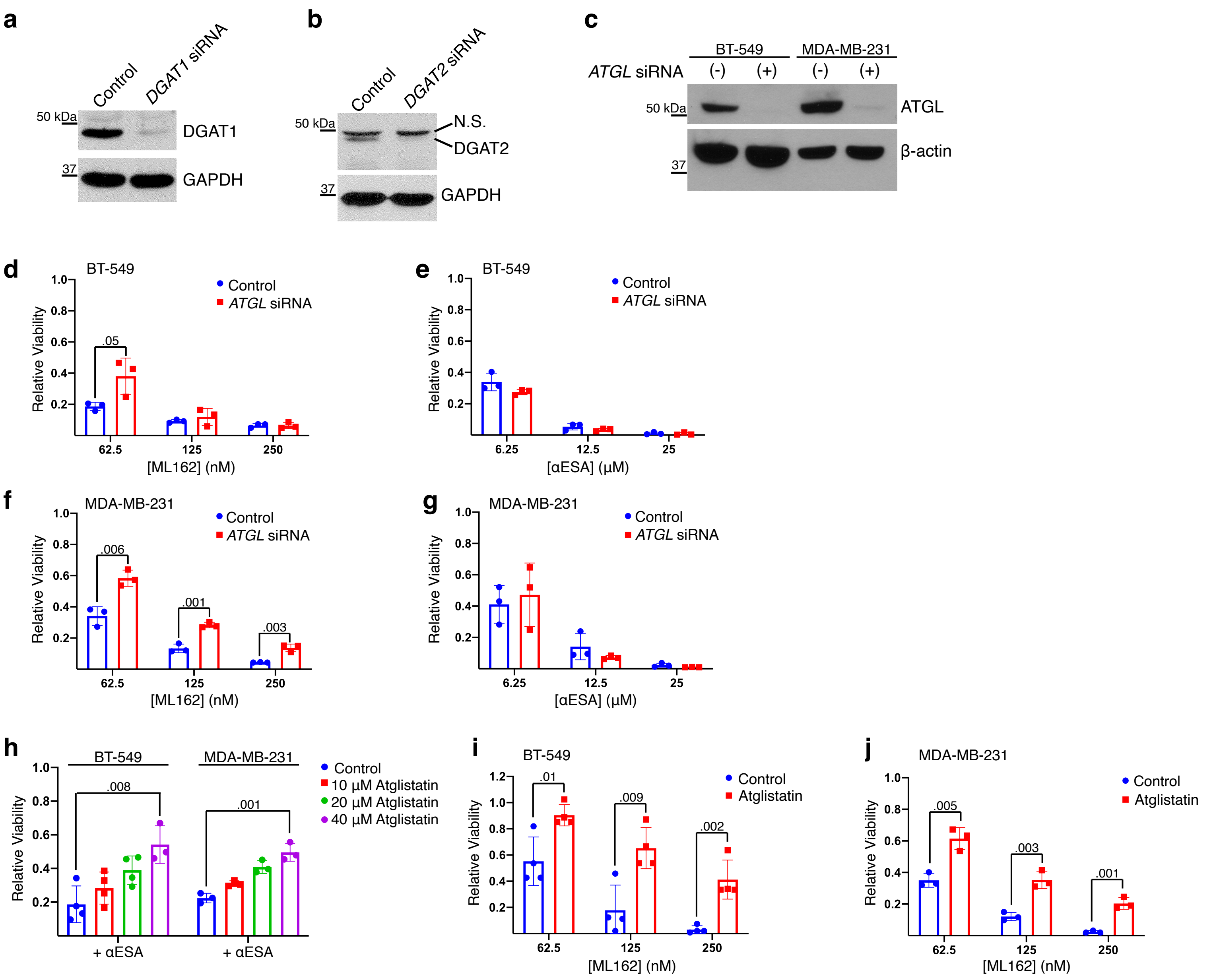

Supplementary Figure 6. **Inhibition of DGAT1 and DGAT2 suppressed *α*ESA toxicity but not ML162 toxicity in BT549 cells. a**, Western blot showing DGAT1 protein level in MDA-MB-231 cells 72 hours after transfection with a pool of *DGAT1*-targeting siRNA or non-targeting siRNA. GAPDH is the loading control. This experiment was performed once. **b**, Western blot showing DGAT2 level in MDA-MB-231 cells 72 hours following transfection with a pool of *DGAT2*-targeting siRNA or non-targeting siRNA. Note that the GAPDH loading control is from replicate lanes distinct from those used to detect DGAT2. The band labeled "N.S." is a non-specific band that can also serve as a loading control. This experiment was performed once. **c**, Western blot showing ATGL protein amount in BT-549 or MDA-MB-231 cells 72 hours after transfection with a pool of *ATGL*-targeting siRNA or non-targeting siRNA. These experiments were performed once. Bar charts showing relative cell viability of BT-549 cells (**d**, **e**) or MDA-MB-231 cells (**f**, **g**) incubated with the indicated dose of *α*ESA or ML162 for 48 hours (n = 3 independent experiments). *α*ESA and ML162 were added 72 hours post-transfection with a pool of *ATGL*-targeting siRNA or non-targeting siRNA. Error bars in these and subsequent panels show standard deviation centered on the mean, and p-values are shown above the comparator bars (two-sided Student's t-test). **h,** Relative cell viability of BT-549 (n = 4 independent experiments for conditions excluding 40 μM atglistatin for which n = 3) or MDA-MB-231 (n = 3) cells incubated for 48 hours with *α*ESA (12.5 μM for BT-549 and 6.25 μM for MDA-MB-231) and either DMSO or the specified dose of atglistatin. Relative cell viability of **i**, BT-549 (n = 4 independent experiments) or **j**, MDA-MB-231 (n = 3) cells treated for 48 hours with the indicted dose of ML162 plus or minus 40 μM atglistatin.

Supplementary Figure 7

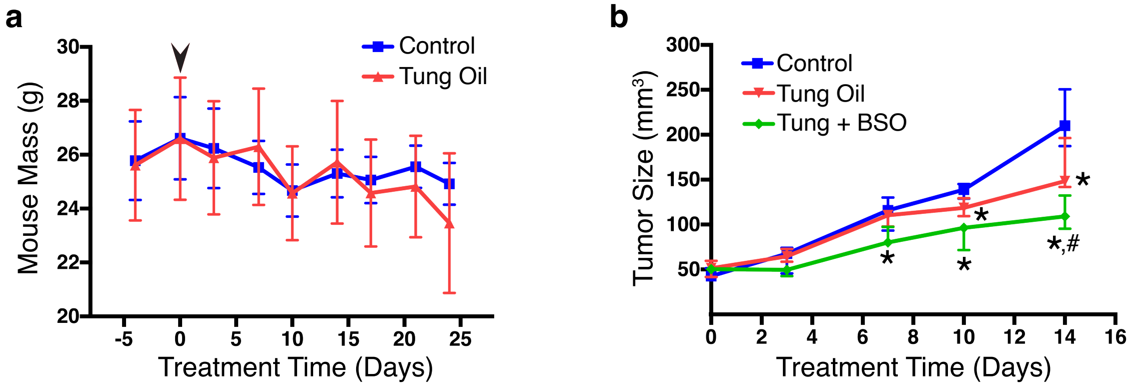

Supplementary Figure 7. **Growth inhibition of MDA-MB-231 xenograft tumors by tung oil was enhanced by BSO.** **a**, Average mass over time for the mice ($n = 6$ per group) included in the experiment shown in Fig. 9a. Error bars show standard deviation centered on the mean. The arrow head marks the start of treatment. **b**, Median tumor volumes for orthotopic MDA-MB-231 xenograft tumors in NSG mice treated for 14 days by oral gavage with either 100 µl safflower oil (control, $n = 6$ mice, bilateral tumors), tung oil ($n = 4$), or tung oil and BSO given at 20 mM in drinking water ($n = 4$). * denotes $p < .05$ compared to the control (two-sided Student's t test). The p-values from left to right are .02 and .006 for tung oil-treated mice, and .009, .0001, and .000003 for tung oil and BSO-treated mice. # indicates $p = .004$ compared to tung oil-treated mice. Error bars show interquartile range.
