# Supplementary material for: Ferroptotic cell death triggered by conjugated linolenic acids is mediated by ACSL1
Source: Nat Commun. 2021 Apr 14;12:2244. doi: 10.1038/s41467-021-22471-y (PMC8046803; doi:10.1038/s41467-021-22471-y)
Supplement: Supplementary file 3 — Description of Additional Supplementary Files [file 41467_2021_22471_MOESM3_ESM.pdf]

Description of Additional Supplementary Files

File Name: Supplementary Movie 1
Description: Time-lapse phase contrast microscopy of BT-549 cells treated with 25 µM aESA at time 0. Elapsed time in hours:min is given within the frame.

File Name: Supplementary Movie 2
Description: Time-lapse phase contrast microscopy of BT-549 cells treated with 0.5 µM ML162 at time 0. Elapsed time in hours:min is given within the frame.

File Name: Supplementary Movie 3
Description: Time-lapse phase contrast microscopy of BT-549 cells treated with 0.5 µM staurosporine at time 0. Elapsed time in hours:min is given within the frame

File Name: Supplementary Data 1
Description: Supplementary Table 1. Lipidomic assessment of aESA incorporation into lipids in MDA-MB-231

File Name: Supplementary Data 2
Description: Supplementary Table 2. Lipidome changes associated with ACSL1 deficiency (vehicle-treated), Supplementary Table 3. Lipidome changes associated with ACSL1 deficiency (aESA-treated), Supplementary Table 4. Comparison of lipidome changes associated with ACSL1 deficiency compared to ACSL1 overexpression, and Supplementary Table 5. Alterations in mole percent of 18:3-containing lipids associated with ACSL1 deficiency.

File Name: Supplementary Data 3
Description: Supplementary Table 6. Lipidomic analysis of oxidized triacylglycerols in BT-549 cells.

File Name: Supplementary Data 4
Description: Lipidomic analysis of oxidized triacylglycerols in BT-549 cells
